# PhenoMiner: from text to a database of phenotypes associated with OMIM diseases

**DOI:** 10.1093/database/bav104

**Published:** 2015-10-27

**Authors:** Nigel Collier, Tudor Groza, Damian Smedley, Peter N. Robinson, Anika Oellrich, Dietrich Rebholz-Schuhmann

**Affiliations:** ^1^The University of Cambridge, Cambridge, CB3 9DB, UK,; ^2^European Bioinformatics Institute (EMBL-EBI), Wellcome Trust Genome Campus, Hinxton, Cambridge, UK,; ^3^Garvan Institute of Medical Research, Darlinghurst, Sydney, NSW 2010, Australia,; ^4^School of ITEE, The University of Queensland, St. Lucia, QLD 4072, Australia,; ^5^Mouse Informatics Group, Wellcome Trust Sanger Institute, Wellcome Trust Genome Campus, Hinxton CB10 1SA, UK,; ^6^Institute for Medical Genetics and Human Genetics, Charité-Universitatsmedizin Berlin, 13353 Berlin, Germany and; ^ 7^The Insight Centre for Data Analytics, National University of Ireland, Galway, Ireland

## Abstract

Analysis of scientific and clinical phenotypes reported in the experimental literature has been curated manually to build high-quality databases such as the Online Mendelian Inheritance in Man (OMIM). However, the identification and harmonization of phenotype descriptions struggles with the diversity of human expressivity. We introduce a novel automated extraction approach called PhenoMiner that exploits full parsing and conceptual analysis. Apriori association mining is then used to identify relationships to human diseases. We applied PhenoMiner to the BMC open access collection and identified 13 636 phenotype candidates. We identified 28 155 phenotype-disorder hypotheses covering 4898 phenotypes and 1659 Mendelian disorders. Analysis showed: (i) the semantic distribution of the extracted terms against linked ontologies; (ii) a comparison of term overlap with the Human Phenotype Ontology (HP); (iii) moderate support for phenotype-disorder pairs in both OMIM and the literature; (iv) strong associations of phenotype-disorder pairs to known disease-genes pairs using PhenoDigm. The full list of PhenoMiner phenotypes (S1), phenotype-disorder associations (S2), association-filtered linked data (S3) and user database documentation (S5) is available as supplementary data and can be downloaded at http://github.com/nhcollier/PhenoMiner under a Creative Commons Attribution 4.0 license.

**Database URL:** phenominer.mml.cam.ac.uk

## Introduction

**Figure 1.**
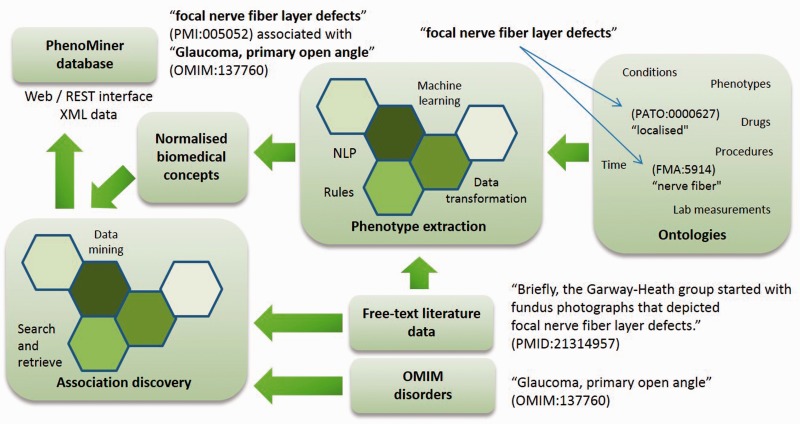
Overview of PhenoMiner illustrating the flow of data from the literature, to text mining, to association discovery and into an integrated semantic representation.

Phenotype descriptions of anatomy, physiology and behaviour such as ‘weak extraocular muscles’ and ‘increased intraocular pressure’ form the basis for determining the existence and treatment of a disease against the given evidence. In recent years, significant effort has been spent to generate standardized phenotypic vocabularies for a variety of organisms [called ‘ontologies’, e.g. Human or Mouse Phenotype Ontologies (1, 2)] and progress has been made to exploit these resources for automatic judgements on the genetic causes of diseases both for human, e.g. Decipher (3) and inferring from animal models (4–6). However, such systems rely on phenotype data that is coded to ontological concepts, database entries or domain-specific nomenclatures. Given further progress in phenotype encoding, we will have clinical and biomedical data resources aligned through phenotypic descriptions and clinicians exploiting the findings from molecular biology for the evaluation of individual genetic dispositions against the differential diagnosis under scrutiny. In this article we contribute to this goal by proposing a novel approach for automatically extracting phenotypes from the scientific literature using text/data mining as shown in Figure 1. Text-mined phenotypes should shorten the work of ontology curators involved in knowledge discovery and integration as well as providing evidence to life scientists and clinicians about phenotype associations with disorders.

**Figure 2.**
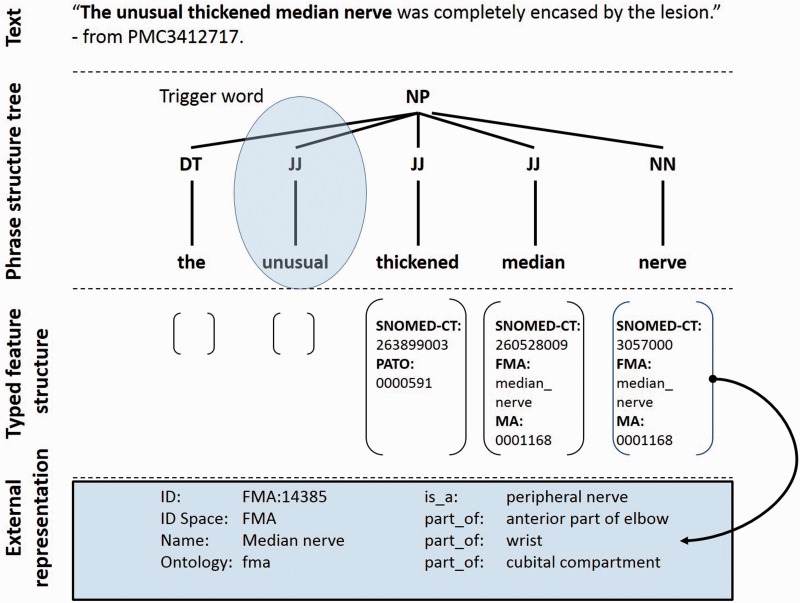
Semantic representation of a text fragment in the PhenoMiner system. Keyword search identifies the potential trigger word *unusual* causing the sentence to be selected for grammatical parsing. The adjectives *thickened* and *median* along with the common noun *nerve* are identified and their corresponding ontology terms are mapped as shown. A semantically typed regular expression then guides the system to select the phrase as a phenotype candidate.

Phenotype descriptions are syntactically and semantically complex because authors exploit the full expressivity of language. Previous computer-based approaches have employed localized patterns, either within a rule-based (7) or machine learning based framework (8, 9). Collier *et al*.’s (10) previous work using a fully supervised approach highlighted the issue of overfitting on a disease domain as well as the fragility of employing the one-class-per-span assumption that is common in named entity approaches. As illustrated in Figure 2, the approach we employ here is the first to explicitly make use of full parsing to create a semantically typed phrase structure tree. By using a tree-matching algorithm the method is capable of handling the disjoint nature of phenotypic mentions, e.g. the separation of *abnormal* and *fourth ventricle* in the relative clause *the fourth ventricle that appeared abnormal*. The approach is also capable of capturing the nested semantics required by phenotypes. We make use of a variety of extant ontologies (e.g. for human anatomical and gene terms) within an entity-quality (EQ) framework to identify phenotype candidates and validate them against disease association profiles in the Online Mendelian Inheritance of Man (OMIM) database (11).

We also deliver a database of phenotype information. The database along with a search box and REST interface and user guide is available from http://phenominer.mml.cam.ac.uk/index.html. The search interface offers stratified refinement of the search result by phenotype, ontology, associated disorder or *P*-value. Each entry contains links to ontologies allowing for a decomposed EQ representation of the phenotype along with PubMedCentral (PMC) indexes to full-text articles and Medline abstracts where evidence about the phenotype occurs. The database covering phenotypes related to abnormal anatomical structures is evaluated intrinsically for quality and coverage against the existing standard in the Human Phenotype Ontology (HP) and extrinsically using data mining for phenotype-disorder associations against a human-curated gold standard in OMIM. Potentially there is a huge space of phenotypes given all of the EQ combinations. This work allows us to begin to answer questions about which forms are actually used in scientific discourse and to harness these for human curation and bioinformatics applications on data in the scientific literature.

## System and methods

### What is a phenotype?

In biology phenotypes are often considered to be observable characteristics of an organism (8), whereas in medical contexts the term *phenotype* is usually considered to denote a deviation from normal morphology, physiology or behaviour (12). This is the working definition that we adopt here and is of particular relevance when considering the profiles of diseases recorded in the free-text literature. In terms of the automated acquisition of phenotypes from text, what makes this task particularly challenging is that it encompasses a range of basic semantic types (e.g. cells, tissues, biological functions) and text types, e.g. scientific texts, clinical trial reports, electronic patient records (EPRs).

### Data sampling

Evidence for phenotype mentions was gathered from the 207 000 document BMC full-text corpus (http://www.biomedcentral.com/about/datamining) using sentences containing a set of context triggers designed to capture abnormalities. We note that triggers which imply more specific abnormalities such as atroph* and hypoplast* will be applied in future studies. The context triggers consisted of the following stems: {abnormal* *|* characteristic*| aberra* *|* defect* *|* atypical* *|* unusual* *|* irregular* *|* anomal* *|* unhealthy *|* inactiv* *|* inadeq*}. The set of triggers was selected based on a core set of synonyms for ‘abnormal’ provided in PATO:0000460. This was then expanded by a computational linguist (NC) using resources such as WordNet and manual analysis of contexts in Medline and EPRs. At this stage we placed no restriction on the domain of the article, so phenotypes may be mined from any organism or type of study. Pruning the set of mined candidate phenotypes to those most relevant for disorders in humans is done later through association rule (AR) mining on the set of OMIM diseases (see Association Data Mining section).

### Text/data mining

Text mining is the application of natural language processing (NLP) to the acquisition of structured information from unstructured texts. Recent use cases include the ShARE/CLEF (13) EPR curation and BioCreative gene curation challenges (14) which provide controlled test suites for system developers.

**Figure 3.**
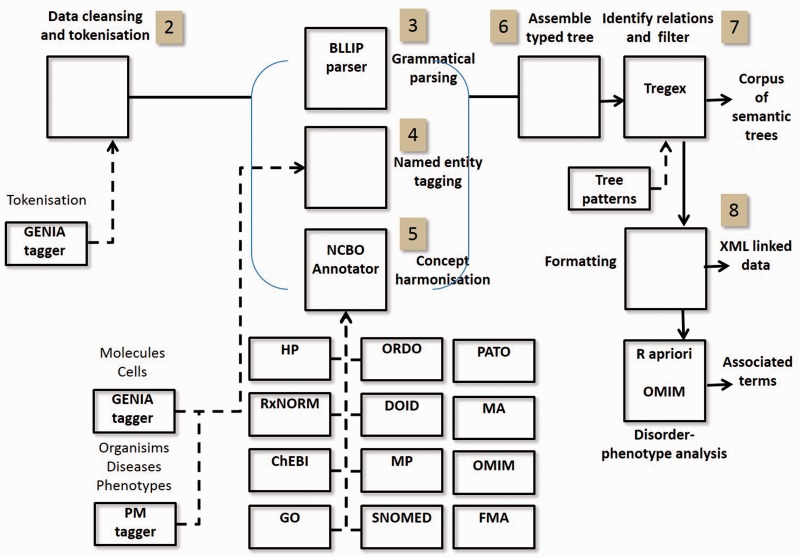
Text/data mining pipeline showing processes and resources. Highlighted indexes correspond to steps in Text/Data Mining section.

The PhenoMiner (PM) system pipeline is outlined in Figure 3. The principal modules are now briefly discussed.

1. Data sampling: As described in Data Sampling section;
2. Data cleansing: split and tokenize the sentences using the GENIA tagger (15) trained on the GENIA Medline abstract corpus;
3. Parsing: phrase structure parsing takes place using the BLLIP/Charniak-Johnson parser (available from https://github.com/BLLIP/bllip-parser); (16) trained on the GENIA corpus as labelled data and PubMed;
4. Named entity recognition: biomedical entities were tagged using thePM NER tagger (17) and the GENIA tagger. This allows us to include semantic labels about anatomical entities, disorders, genes, proteins and other entities that might not be matched in the external vocabularies of the NCBO Annotator.
5. Concept annotation: text spans corresponding to concepts in external vocabularies were recognized using the NCBO Annotator (18) and their links recorded. See Concept Annotation section;
6. Tree assembly: using the phrase structure parse tree as a frame, the lexical leaf nodes were transformed to encode the semantic features extracted from earlier stages;
7. Relation identification: phenotypes were extracted based on a set of patterns encoded in Stanford Tregex (19);
8. Data formatting: the raw data from phenotype identification was transformed into XML using custom Perl scripts that encoded the term, its identifier, the date when the annotation was made, links to external vocabularies, associated OMIM disorders (found from Apriori associations) and links to occurrences of the phenotype in the literature.

The pipeline was implemented and run on a twenty-four 2.66 GHz Xeon core server running on Ubuntu Linux 9.04. Various programming languages are used to implement the algorithms with process flow control scripts in Perl.

### Concept annotation

Free-text phenotypes require disambiguating in order to make clear the similarity between different surface forms. Harmonization (also called ‘normalization’) can be approached by decomposing the free-text phrase and mapping to unique identifiers in external semantic resources such as ontologies. The task is complex and challenging because the same free-text phrase can refer to different ontological concepts in different contexts (e.g. *high blood pressure* as either *Elevated diastolic blood pressure* (HP:0005117) or *Elevated systolic blood pressure* (HP:0004421) or *Hypertension* (HP:0000822)). A variety of techniques have been employed such as heuristics (e.g. choosing the most common sense), comparing matching similarity on the words in the phrase, using local contextual features, acronym expansion, as well as more complex techniques such as topical coherence.

Because of its stability and wide range of underlying ontologies we chose to use the National Center for Biomedical Ontology (NCBO) Annotator. NCBO Annotator is available as a Web service that exploits regular expression matching to identify heterogeneous biomedical concepts in a range of ontologies in the Unified Medical Language System (UMLS) (20) and BioPortal (21). We chose several that are of particular relevance to the composition of phenotypes such as the HP (1), RxNORM (22), ChEBI (23), Gene Ontology (GO) (24), the Phenotypic Attribute and Trait Ontology (PATO) (25), Mouse Adult Gross Anatomy Ontology (MA) (26), the Human Disease Ontology (DOID) (27), Mammalian Phenotype Ontology (MP) (2), SNOMED CT (28), OMIM (11) and the Foundation Model of Anatomy (FMA) (29).

### Tregex

Because phenotype mentions occur in a variety of syntactic environments many different patterns are required to capture them. Traditional regular expressions are fragile for capturing terms with disjoint parts and require substantial effort to develop and post-filter. As evidenced by shared evaluations such as BioNLP (30) we believe that full parsing technology is now robust enough to handle the scientific literature.

**Figure 4.**
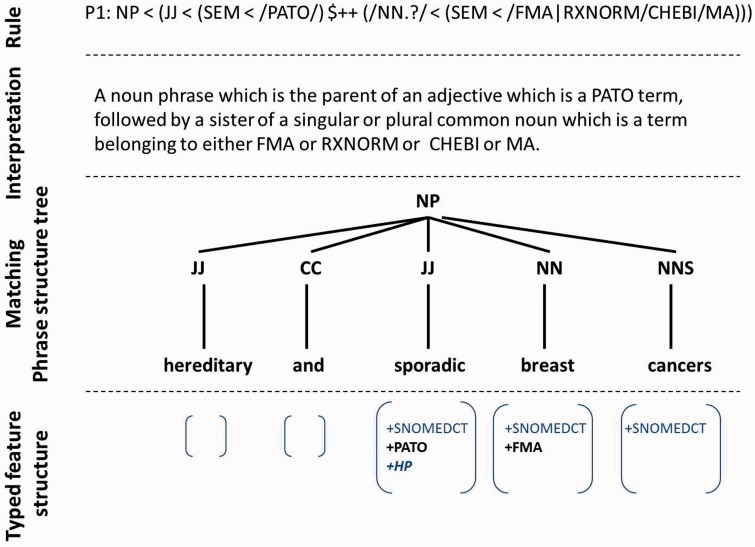
Example of semantic tree matching with a Tregex rule.

In these experiments we have explored syntactic patterns that occur in a context of abnormal phenotype morphology. This led to 134 939 phenotype phrase structure trees being mined. Below is a list of the patterns in Stanford Tregex form that capture these phenotypes. Patterns were hand crafted based on a set of semantic frames focused on PATO modifiers and careful analysis of examples in the literature. Lexical constraints on the leaf nodes have been replaced with regular expressions for matching to semantic constraints on the set of predicated ontology categories (SEM) attached to each word in the sentence, e.g. *PATO* corresponds to a word that is linked to a PATO concept. Each pattern is followed by an illustration of the type of text fragment which it matches (the surrounding text has been omitted for brevity). An illustrative example is shown in Figure 4. Note that non-leaf labels correspond to grammatical phrase structure categories such as *DT* for determiner, *NN.?* for any type of noun, *JJ* for adjective and *CC* for a conjunction. Future work will look at comparing our hand-crafted Tregex patterns to patterns discovered using machine learning.

1. *NP < (JJ < (SEM < /PATO/)* ++ *(/NN.?/ < (SEM </FMA|RXNORM|CHEBI|MA/)))*. e.g. a large dilated esophagus. In this pattern we are filtering for noun phrases (NP) that contain a child (*<*) adjective (JJ) which matches to a PATO concept. The adjective should have a sister (++) noun (NN.?) that matches to a concept in FMA, RxNORM, CHEBI or MA.
2. *NP < ((ADJP < (JJ < (SEM < /PATO/)))* ++ *(/NN.?/ < (SEM </FMA|RXNORM|CHEBI|MA/)))*, e.g. abnormally long polyglutamine tract
3. *NP < ((NP < (JJ < (SEM < /PATO/)))* + *CC (NP < (NN < (SEM< /FMA|RXNORM|CHEBI|MA/))))*, e.g. severe burns and solid organ injuries
4. *NP < (JJ < (SEM < /PATO/)* ++ *(/NN.?/ < (SEM < /MP|HP/)))*, e.g. abnormal tongue position
5. *NP < ((ADJP < (JJ < (SEM < /PATO/)))* ++ *(/NN.?/ < (SEM </MP|HP/)))*, e.g. a very short life span
6. *NP < ((NP < (JJ < (SEM < /PATO/)))* + *CC (NP < (NN < (SEM </MP|HP/))))*, e.g. abnormal dentition and delayed tooth eruption
7. *NP < (JJ < (SEM < /PATO/)* ++ *(/NN.?/ < (SEM </DOID|ORDO|OMIM/)))*, e.g. right quadrant pain
8. *NP < ((ADJP < (JJ < (SEM < /PATO/)))* ++ *(/NN.?/ < (SEM </DOID|ORDO|OMIM/)))*, e.g. intrapartum/early neonatal death
9. *NP < ((NP < (JJ < (SEM < /PATO/)))* + *CC (NP < (NN < (SEM </DOID|ORDO|OMIM/))))*, e.g. slow healing and excessive scaring

### Formatting

Since many phenotypes occur only rarely, we decided to keep only those 9792 candidates that have a document frequency (df) of 10 times or more in a search of PMC (abstracts and full texts) using the phenotype term in the E-utilities Web interface. In total 12 747 phenotype candidates (93.5%) returned one or more PubMed identifiers (PMIDs). 8033 (58.9%) had df* ≥ *25. 281 phenotype candidates (2.1%) had df* ≥ *10 000. Examples of very low document frequency phenotypes in PMC include *heritable inverted teat defect*, *multiple platelet defects* and *prominent atypical cannabinoid receptor*. High-frequency phenotypes include *abnormal liver function behaviour* and *heart defects*. In general low-frequency phenotypes are typically quite long and might contain descriptive qualifiers, conjunctions or lists. For example, (i) *prominent midline neural tube defect* (df = 6) and *neural tube defect* (df = 1186), and (ii) *cautious abnormal gait strategies* (*d*f = 2), *cautious abnormal gait* (df = 5), *abnormal gait strategies* (*d*f = 62) and *abnormal gait* (*d*f = 504). The full list of 9792 extracted phenotypes with df* ≥ *10 along with linked concept identifiers and PMIDs is contained in Supplementary material S1.

### Association data mining

In order to find significant associations between phenotype candidates and disorder terms we chose to use literature indexing as our intermediate feature set. Mining term associations using topical annotations has a long history, (31) who looked at evidence to support a therapeutic relation between *Curcumin Longa* and retinal diseases based on MeSH profiles in the literature. In our case we wanted to know whether there is association-based evidence for known or possibly novel phenotype-disease relations that can be verified against human curated standards.

*Disorder data:* Synonyms play an important role in identifying disorder concepts. For example ‘Leopard syndrome’ can include synonyms such as ‘Gorlin syndrome’, ‘Cardiocutaneous syndrome’ and ‘Lentiginosis profusa syndrome’. Query expansion in this way increases the available evidence considerably. We chose the list of OMIM disorders and their synonyms provided by the Merged disease vocabulary (MEDIC) (32, 33) which unifies OMIM terms with Disease subtree of the National Library of Medicine’s Medical Subject Headings (MeSH). We used the July 2014 build (http://ctdbase.org/down loads/) and selected all terms with an OMIM alternative disease identifier. From these we created an intermediate file consisting of the disease name and all synonyms which we searched for in PMC E-utilities using the same query syntax for phenotypes.

All PMIDs that were returned were unified by labelling with the canonical disease name in MEDIC (e.g. ‘Leopard syndrome’). For individual search terms we restricted the search to return 10 000 PMIDs but because of the synonym expansion the cumulative totals (illustrated below) were higher in some cases.

In total 2885 disorders returned one or more PMIDs. 2426 disorders (84%) had df* ≥ *25 623 disorders (22%) had a cumulative df* ≥ *10 000. Examples of very low-frequency disorders in PMC include *Bothnia retinal dystrophy*, *congenital preauricular fistulae* and *Joubert syndrome 6*. High-frequency disorders include *Schizophrenia*, *Hodgkin disease* and *Type 2 diabetes mellitus*.

*Apriori:* After discovering phenotype candidates we post-filtered them by frequency of association with human disorders. Motivated by the studies of association mining concept relations (34) and (35), we explored the use of the R *Apriori* algorithm (36) for identifying disorder-phenotype rules. AR mining attempts to discover rules between frequently co-occurring items in a transaction data set. A typical use case scenario is discovering products that are often purchased together. In our case we want to discover rules of the form *P → D*, where *P* (the *antecedent*) is drawn from the set of phenotypes we discovered previously, and *D* (the *consequent*) is drawn from the set of OMIM disorders. The set of OMIM disorders and their synonyms was obtained from MEDIC (32). PMIDs are used to label the transaction items and are found for each phenotypes and disorder by querying the PMC E-utils RESTful Web Service with the phrase or the disorder in the ‘term’ field and ‘retmax’ set to 10 000.

In contrast to our approach, Razan *et al.* [34] investigated AR mining on EPRs and focused on 114 skeletal dysplasias. Using NCBO Annotator with HP as the target ontology they looked at identifying the set of phenotypes for a disorder, i.e. *pathognomonic* features. They applied a novel association mining algorithm with measures of commonality and confidence in place of standard support and confidence. In future work we would like to compare *Apriori* to their method using our data.

The input to *Apriori* is a database containing a list of transactions, each with a unique PMID identifier. For example one transaction was obtained with PMID 18 852 161 containing the terms *{d/Marfan Syndrome, p/abnormal chest signs, p/abnormal connective tissue, p/abnormal connective tissue structure, p/abnormal tissue pathology, p/abnormal weight control}*, where the suffix d denotes a disorder and p denotes a phene.

We applied *Apriori* using the following parameters to obtain a set of meaningful ARs: sup=0.00000025, conf=0.1, minlen=2, maxlen=2, target=‘rules’. A further threshold was a minimum document frequency of 10 for phenotypes. Where, (i) *sup*: is the minimum support for an itemset (e.g. *{*d/Marfan Syndrome, p/abnormal connective tissue*}*)). With approximately 7 million item sets over 12 675 items, support corresponds to a restriction of two documents containing the phene-disorder association. This low level was set to capture as wide a range of associations as possible and to allow users of the data to apply their own filtering on the data. The mean and median support were 1543 and 20 document associations respectively.

Maximum support was for 30 000 document associations. (ii) *conf* is the minimum confidence for rules and corresponds to the conditional probability of the disorder given the phene, e.g. P(d/Marfan Syndrome*|*p/abnormal connective tissue). The mean and median confidence were 0.34 and 0.20, respectively. Maximum confidence was 1.0. (iii) *minlen* is an integer value for the minimum number of items in the rule set, i.e. the minimum cardinality of the rule set, (iv) *maxlen* is an integer value for the maximum number of items in the rule set and (v) *target* is a character string indicating the type of association to be mined. The mined rules are post-filtered so that only those of type *{phenotype} → {disorder}* are retained. *P* values for each of the extracted rules were used to allow ranking of the ARs. These are calculated using Fisher’s exact test on the contingency table within *Apriori* and are provided in Supplementary materials S2 and S3.

### Experimental setup

This study provides a quantitative and a qualitative assessment of the mined phenotypes. The goal of the quantitative assessment was (i) to characterize the mappings of PM terms according to a range of biomedical ontologies; (ii) to evaluate the coverage of PM terms with respect to the human-curated gold standard in HP using the Bio-LarK concept recognition system (http://biolark.org/) and to stratify this coverage by affected anatomical system in the HP concept hierarchy; (iii) to evaluate the quality of phenotype-disorder associations from *Apriori* against the OMIM database (http://omim.org/) gold standard; (iv) to benchmark the quality of PM terms against HP terms for discovering known gene-disorder associations. Evaluation was performed by PhenDigm (6), a system for semantic mapping of clinical features observed in humans and mouse.

## Results

Using the text-mining pipeline we were able to extract 13 636 phenotype terms from the BMC collection. These were filtered in two stages: first we searched for the terms in PMC and removed any term with a document frequency of less than 10. Then we applied AR mining on the set of remaining terms and a collection of disorder terms from OMIM (described later). We retained a set of 4898 terms that were found to be highly associated with disorders available in Supplementary material S2. These terms were then formatted in XML and are provided along with semantic links to the ontologies described in Concept Annotation section as well as PMIDs to full-text and Medline abstracts from the PMC Web service (available in Supplementary material S3).

### Quantitative evaluation of PM concept mappings

Table 1 shows the distribution of mappings to external vocabularies found by NCBO Annotator. The results however represent approximate values due to factors such as unmatched synonyms or unrecognized word ordering (false negatives) and incorrectly resolved polysemy (false positives).

**Table 1.** Representation of external concepts in disorder phenotype terms

Ontology (O)	*P*(T,O)	Ontology (O)	*P*(T,O)
PATO	0.99	MP	0.24
SNOMEDCT	0.98	ORDO	0.21
OMIM	0.96	MA	0.15
HP	0.57	RxNORM	0.09
FMA	0.44	ChEBI	0.02
DOID	0.30	GO	0.00

Probability that an extracted Phenotype term (T) will have an ontology concept (O) associated with it based on NCBO Annotator data (18). Total number of terms is 4898.

Because all the Tregex patterns required a PATO entity, it is not surprising to find a high incidence of these terms. Moreover, given the coverage and granularity of SNOMED CT, it is also unsurprising to find high coverage there. HP and FMA are well represented with nearly half of our candidates having a mapping to some concept (see manual analysis in Quantitative Evaluation of HP Coverage section). It is encouraging to find a moderate number of mappings to rare disorders in DOID and ORDO. The very low number of mappings to GO probably reflects the focus of the context trigger words on anatomic structures rather than processes.

### Quantitative evaluation of HP coverage

PM terms and HP concepts have been automatically aligned using a second concept recognition system called Bio-LarK (37). Bio-LarK uses an information retrieval approach to index and retrieve HP concepts, combined with a series of linguistic techniques to perform term normalization and decomposition (e.g., token lexical variation). In addition to standard concept recognition, the system is able to decompose and align conjunctive terms (e.g. *short and broad fingers* aligned to HP:0009381 *Short fingers* and HP:0001500 *Broad fingers*), as well as recognize and process non-canonical phenotypes, such as *fingers are short and broad* which would be aligned to the same terms as in the previous example. Bio-LarK has been extensively tested and achieved 86.2% *F*-Score on a set of 2075 manually crafted HP test suites that included a varied range of tests from length-based cases to canonical vs. non-canonical ordering, coordination or synonymy.

Using the set of 4898 terms we were able to obtain a successful complete or partial HP mapping against 10 900 HP terms for 2254 PM terms. The complete set of inferred HP maps from Bio-LarK are provided within Supplementary material S3. By comparing the PM to HP mappings with the HP concepts, we were able to obtain data for coverage of PM at the system level. The results are presented in Table 2. We can see from the data that PM terms from the BMC open access collection have greatest coverage in HP’s *Abnormality of breast* category. This aligns with our expectations due to the high volume of breast cancer research reported in the collection. The results in Table 2 need to be interpreted with some caution because HP has a high proportion of concepts in abnormality of the skeletal system (28.8%) due to the growth in anatomical partonomy relations (e.g. *phalanx of finger of hand*). Whilst the coverage in PM is low, the mined terms might provide insights to guide curators in which anatomical partonomy phenotypes are actually mentioned by authors.

**Table 2.** HP coverage

Affected system	ID	%
Abnormality of the endocrine system	HP:0000818	9.0
Abnormality of prenatal development or birth	HP:0001197	6.7
Neoplasm	HP:0002664	17.3
Abnormality of the respiratory system	HP:0002086	14.1
Abnormality of the genitourinary system	HP:0000119	13.2
Abnormality of the nervous system	HP:0000707	12.6
Abnormality of the musculature	HP:0003011	7.0
Abnormality of metabolism/homeostasis	HP:0001939	8.4
Abnormality of blood and blood-forming tissues	HP:0001871	15.2
Abnormality of the immune system	HP:0002715	15.9
Abnormality of the voice	HP:0001608	13.3
Abnormality of the skeletal system	HP:0000924	2.7
Abnormality of the ear	HP:0000598	5.7
Abnormality of head and neck	HP:0000152	8.4
Abnormality of the breast	HP:0000769	17.4
Abnormality of the integument	HP:0001574	6.5
Growth abnormality	HP:0001507	15.7
Abnormality of the abdomen	HP:0001438	14.6
Abnormality of the cardiovascular system	HP:0001626	12.5
Abnormality of the eye	HP:0000478	8.0
Abnormality of connective tissue	HP:0003549	6.2

Percentage overlap between PhenoMiner terms and HPO terms estimated using Bio-LarK’s concept alignment.

### Qualitative evaluation of PM terms

Manual analysis of the PM to HP mapping process by the authors revealed several categories of challenges: (i) Missing terms: as hoped PM discovered many phenotype terms that appeared to be novel for HP. These include *abnormal neural plate morphology* (PM1029) and *abnormal neuron excitability* (PM1031). Such terms included complex forms involving biological processes such as *abnormal neutrophil oxidase function* (PM1035). They also include synonyms, e.g. *malformed inner ears* (PM5543) which is a non-specific term closely related to *morphological abnormality of the inner ear* (HP:0011390). The missing terms also raised some interesting thoughts about how qualifiers should be applied to existing complex concepts such as *obesity* (HP:0001513), as in *abnormal obesity* (PM1045) and *anxiety* (HP:0000739) as in *abnormal anxiety* (PM102); (ii) Coordination: PM terms containing coordinated entities need to be decomposed to understand whether they are individual or complex terms. e.g. *abnormal myocardial perfusion and obstructive coronary disease* (PM1014). In some cases a complex term was already registered in HP, e.g. *acute fasting hypoketotic hypoglycaemia* (PM1822) as HP:0001943, and in others Bio-LarK successfully formed a union of two extant HP terms, e.g. *rapidly progressive visual field defects* (PM7632) as *progressive visual field defects* (HP:0007987) and *rapidly progressive* (HP:0003678); (iii) Acronyms: abbreviations were not explicitly handled during term mining and require disambiguation/expansion as part of future work, e.g. *abnormal ogtt and high insulin* (PM1046) which would map to *abnormal oral glucose tolerance test* (HP:0004924); (iv) Qualifiers: some terms in HP are handled in a de-compositional manner, e.g. *abnormal depression* as *abnormal* + *depression* (HP:0000716)) whilst some are handled in a pre-compositional manner, e.g. *abnormality of cardiovascular system physiology* (HP:0011025); (v) Post-nominal modifiers: phenotypes may participate in other terms, e.g. *abnormal nerve conduction studies* (PM1019), requires the exclusion of *studies* to map to *abnormal nerve conduction* (HP:0000762); (vi) World view: we found some cases where the semantic view between the mined terms and the HP terms differ, e.g. *abnormal nocturnal blood pressure* (PM1038) could not be mapped to either *elevated blood pressure* or *low blood pressure*; (vii) Missing context: as a final category of challenges we found that in some cases extra context was required to disambiguate and achieve a correct alignment from PM to HP. For example, *absent pituitary* (PM1571) is missing information about ‘anterior’ or ‘posterior’, and *abnormal overgrowth* (PM1061) is missing a subject.

### Quantitative evaluation of PM-OMIM associations

Three experienced biomedical experts, each with over 10 years’ experience in biomedical genetics were asked to rate the phenotypes, disorders and relations. The experts were encouraged to pay reference to their intuitions, OMIM and the scientific literature. Sampling chose 200 phenotype-disorder associations from the high confidence pairings given in the Supplementary material S2 at ranks 1–40, 101–140, 201–240, 401–440 and 501–540.

Each phenotype, disorder and association was scored as either *incorrect*, *possibly correct* or *correct*. For example, *low red blood cell count* (anemia) was found to be associated with Gaucher Disease and *abnormal cytokine expression* was found to be associated with Acute Myeloid Leukemia. To simplify agreement calculations, correct and possibly correct judgements were conflated into a single positive judgement. Phenotype terms were judged to be positive if they (i) denoted a deviation from normal morphology, physiology or behaviour, (ii) were sensible English and (iii) were used in the literature. Disorders were positive if they denoted an OMIM disorder and were sensible English. Paired associations were positive if either (i) the disease led to the phenotype or (ii) the disease led to secondary complication that included the phenotype. One annotator was chosen who had the most experience in phenotype curation (PR) and the other two annotators’ judgements were used to calculate inter-coder (i.e. ‘inter-rater’) agreement. It should be noted that of the three annotators, PR had the lowest number of *possibly correct* judgements for associations. Using Wilson score intervals the rate of positive judgements for phenotypes (*n* = 200) was calculated as 0.68 with [0.61,0.74] 95% confidence intervals (95% CI). This compares to a macro-average positive judgement of 0.62 for all three annotators. Positive disorders (*n* = 200) were found to be almost totally correct (1.0) with [0.99, 1.0] 95% CI. Finally positive judgements on phenotype-disorder associations (*n* = 200) were calculated at 0.38 with [0.32,0.45] 95% CI. This compares to a macro-average of 0.45 for all three annotators.

Inter-coder agreement (38) between PR and the other two annotators was calculated for phenotypes (*n* = 200) using pairwise agreement (70% and 80%) and Cohen’s Kappa (0.34 and 0.59) which can be interpreted as *Moderate*. Pairwise agreement on the disorders (*n* = 200) was almost unanimous (*>*99%).

Pairwise agreement on the phenotype-disorder associations (*n* = 200) was 64% and 65% with Cohen’s Kappa of 0.24 and 0.32 which is only *Fair* on the scale reported in Artstein and Poesio. When we selected only phenotypes where PR and each of the other coders had a consensus opinion, pairwise agreement for phenotype-disorder associations rose to 74% (+10%, *n* = 152) and 70% (+5%, *n* = 138) with Cohen’s Kappa of 0.48 (+0.24) and 0.45(+0.13) which is *Moderate*.

When we looked in detail at the incorrect phenotypes, we found several causes of error: (i) Incomplete phrases, e.g. *the other 50*, *slow progression* and *unusually long*. These candidates generally consisted of a quality but lacked an entity; (ii) Non-specific phenotypes, e.g. *abnormal molecular weight* and *advanced neoplasia*. These candidates were missing a specific entity in which the quality inheres; (iii) Structure not phenotypes, e.g. *closed chromatin structure* and *tripartite motif protein 5*. These candidates on the surface had both qualities and entities but were descriptive names of structures; (iv) Disorder not phenotypes, e.g. *recurrent staphylococcal and candidal skin infections*; (v) Coordinate phrase containing a phenotype, e.g. *detecting and treating abnormal blood sugars*. Here we observed that one of the coordinating terms was a phenotype whilst the other was not; (vi) Other errors, e.g. *offset variable* and *terminal differentiation and ongoing cell proliferation*. A manual analysis was conducted on the full set of automatically discovered phenotypes (Supplementary material S3) to remove 373 erroneous terms. The resulting set of terms was re-indexed using the identifier ‘PMI’ and is now available as Supplementary material S6.

### Quantitative evaluation of PM-OMIM associations by known-gene associations

In previous work on PhenoDigm, we have shown that the manually curated OMIM HP annotations can be used in cross-species semantic comparisons to mouse mutant phenotypes annotated using the Mammalian Phenotype Ontology (MP) (6). We were able to recall known gene-disease associations described in OMIM’s MorbidMap and Mouse Genome Database (MGD) literature curated mouse models of human disease with high specificity and sensitivity. To establish whether the PM annotations are also able to be used in such comparisons we repeated this assessment for the 1,234

OMIM diseases that contained one or more PM annotations that could be mapped to HP. The performance in recalling known disease gene associations using mouse models was compared with that when using the manually curated HP annotations for these 1234 diseases.

**Figure 5.**
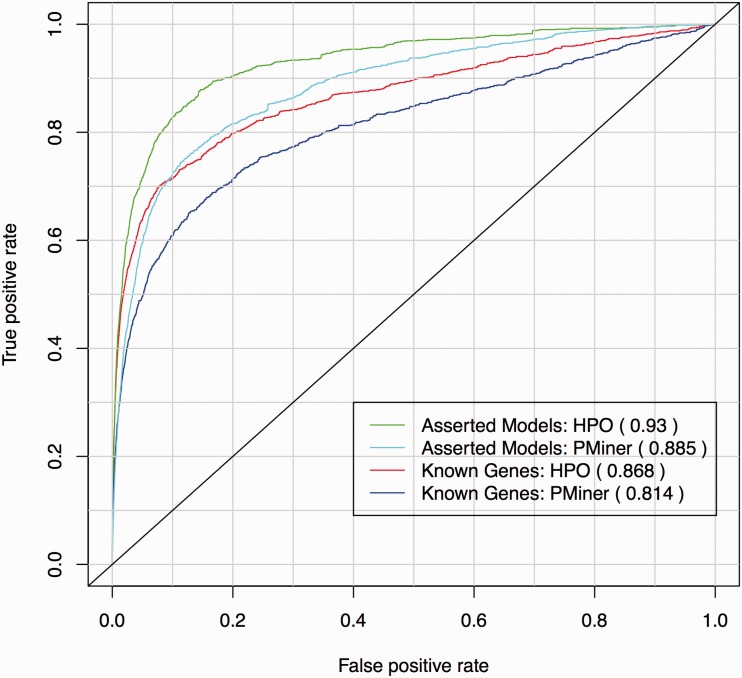
ROC curve for known gene-disease associations from OMIM’s MorbidMap using HP and PhenoMiner annotations. For each disease the true- and false-positive rate is calculated from the list of mouse genes in MGD ordered by phenotypic similarity and the known associated gene in OMIM.

The performance of the method was captured in a receiver operating characteristic (ROC) curve, which is shown in Figure 5. For each disease, the true- and false-positive rate are calculated from the list of mouse genes ordered by phenotypic similarity and the known associated gene. The ROCR R package was used to calculate and plot these values for each disease and determine the area under the curve (AUC), specificity and sensitivity. In ROC analysis, obtaining an AUC score in a range of 0.5-1 indicates that the applied prioritization algorithm is valid, and the predictions conform to the existing biological knowledge, with the higher the value, the better the fit. The AUC of known disease genes from OMIM was notable for both the manually curated HP annotations and the PhenoMiner annotations (0.868 and 0.814, respectively) with the performance of PhenoMiner’s annotations only slightly below that seen for the manual curations. Given that many of the additional PhenoMiner annotations could not be mapped to HP and were therefore not available for this experiment, this represents an impressive validation of PhenoMiner. The results for the MGD manually asserted models were similar with PhenoMiner giving an AUC of 0.885 compared with 0.93 for manually curated HP annotations.

## Conclusion

The combinatorial nature of phenotypes that occurs through the composition of various semantic types makes capturing them a major challenge. Taken together the tests we have done indicate that our methods can successfully capture a range of human phenotypes from the scientific literature that are relevant for a wide range of heritable diseases. Our approach provides a novel technique for flexibly capturing diverse phenotypes and allows us to begin to answer the question about which phenotype *forms* are actually used in the scientific literature and how frequently. Furthermore we have begun to make progress towards automated extraction of phenotype profiles for human Mendelian diseases and cross-species comparison.

The current method of exploiting literature-level cooccurrence between a phenotype and a disorder works well in achieving its goal but understanding the fine-grained relationship that is being reported is key to helping curators make the final decision about inclusion of the phenotype in ontologies like the HP. This is an area we have already started and will be reporting in future publications.

Our systematic comparison of the captured phentoypes revealed interesting trends in the systems covered by the full-text articles in the BMC open access collection and their overlap with expert curated phenotypes in the HP. In future work we intend to provide an expert curation study of those phenotypes not matched to HP and we will look at expanding the groupings of from structural abnormalities to increase coverage.

## Supplementary data

Supplementary data are available at *Database* Online.

Supplementary Data
