# Supplementary material for: PhenoMiner: from text to a database of phenotypes associated with OMIM diseases
Source: Database (Oxford). 2015 Oct 27;2015:bav104. doi: 10.1093/database/bav104 (PMC4622021; doi:10.1093/database/bav104)
Supplement: Supplementary Data [file supp_2015_bav104_index.html]

Supplementary Data 

# PhenoMiner: from text to a database of phenotypes associated with OMIM diseases

## Supplementary Data

files

- Supplementary Data - pdf file
- Supplementary Data - pdf file
