# Supplementary material for: PhenoMiner: from text to a database of phenotypes associated with OMIM diseases
Source: Database (Oxford). 2015 Oct 27;2015:bav104. doi: 10.1093/database/bav104 (PMC4622021; doi:10.1093/database/bav104)
Supplement: Supplementary Data [file supp_bav104_S5.pdf]

PhenoMiner Web Search and REST guide

VERSION 1.1 (July 2015)

Authors: Nigel Collier[1,2*] and Vu Tran Mai[3]

[1] University of Cambridge, Cambridge, UK

[2] European Bioinformatics Institute, Hinxton, Cambridge, UK

[3] University of Engineering and Technology – VNU, VietNam

[*] To whom correspondence should be addressed

SCOPE NOTE

This report outlines the search interface and REST interface for the PhenoMiner database.

LICENSE

This work is licensed under a Creative Commons Attribution 4.0 International License.

CONTACT

For further information about this technical report, please contact:

Nigel COLLIER

Department of Theoretical and Applied Linguistics

University of Cambridge

9 West Road, Cambridge, UK

PhenoMiner Web Search and REST Guide

Overview

Phenotypes play a key role in inferring the complex relationships between genes and human heritable diseases. Analysis of scientific and clinical phenotypes reported in the experimental literature has been curated manually to build high quality databases such as the Online Mendelian Inheritance of Man (OMIM). However, the identification and semantic harmonisation of phenotype descriptions is a time consuming process that struggles to come to grips with the diversity of human expressivity. High throughput text mining, enhanced with automated conceptual analysis now make it possible to identify phenotype mentions and to predict associative relationships with diseases. We show the effectiveness of our approach by comparing the results against the manually curated gold standards in the Human Phenotype Ontology (HPO) and the phenotype-disorder relations in OMIM.

Following a series of experiments we have applied text/data mining to extract and filter a set of phenotype candidates and link these to associated concepts and literature references. We now wish to make these available as a database and shared portal. The data and experiments are being written up and made available through various means – as journal and conference publications, as a downloadable XML database (through GitHub at https://github.com/nhcollier/PhenoMiner and CERN's Zenodo at DOI: 10.5281/zenodo.12493), as literature annotations (via EMBL-EBI's External Links service) and as a standalone demonstration database portal and REST interface. The last of these will be outlined in this document. The Web-GUI is available via: http://phenominer.mml.cam.ac.uk/index.html and the REST interface is available from: phenominer.mml.cam.ac.uk:8080/phenominer/phenotype/_search?q=

Document Data Type (DTD) definition

The XML data file available for download from GitHub and Zenodo (DOI: 10.5281/zenodo.12493) contains text mined evidence about phenotypes. The evidence in the first release is gathered from mining the BMC open access full text collection and then verifying automatically using disease-phenotype associations across all of the PMC literature. The following DTD describing the formatted data we have mined:

```
<!ELEMENT annotationCollection (Term*)>
<!ELEMENT Term (qualifierList,Link*,Tree,associatedDisorders?,fullTextList,abstractList)>
<!ELEMENT qualifierList EMPTY>
<!ELEMENT Link EMPTY>
<!ELEMENT Tree (#PCDATA)>
<!ELEMENT associatedDisorders (disorder+)>
<!ELEMENT disorder (name,omim_id)>
<!ELEMENT name (#PCDATA)>
```

```
<!ELEMENT omim_id (#PCDATA)>
<!ELEMENT fullTextList (Id*)>
<!ELEMENT abstractList (Id*)>
<!ELEMENT Id (#PCDATA)>

<!ATTLIST Term ID CDATA #REQUIRED>
<!ATTLIST Term KEY CDATA #REQUIRED>
<!ATTLIST Term EVIDENCE CDATA #REQUIRED>
<!ATTLIST Term DATE CDATA #REQUIRED>
<!ATTLIST Link text CDATA #REQUIRED>
<!ATTLIST Link ontology CDATA #REQUIRED>
<!ATTLIST Link ID CDATA #REQUIRED>
<!ATTLIST Link evidence CDATA #REQUIRED>
<!ATTLIST associatedDisorders source CDATA #REQUIRED>
<!ATTLIST associatedDisorders min_supp CDATA #REQUIRED>
<!ATTLIST associatedDisorders min_conf CDATA #REQUIRED>
<!ATTLIST associatedDisorders df CDATA #REQUIRED>
<!ATTLIST disorder supp CDATA #REQUIRED>
<!ATTLIST disorder conf CDATA #REQUIRED>
<!ATTLIST disorder lift CDATA #REQUIRED>
<!ATTLIST disorder pval CDATA #REQUIRED>
<!ATTLIST fullTextList source CDATA #REQUIRED>
<!ATTLIST fullTextList df CDATA #REQUIRED>
<!ATTLIST fullTextList retmax CDATA #REQUIRED>
]>
```

The elements and attributes are now described in detail:

Element: Term

The data in this required element describes one complete phenotype term. There is no effort at this stage to unify or encode synonyms so different forms (e.g. plurals) might appear as distinct terms.

Attributes for Term include:

ID This is the surface form of the phenotype term as it appears in text

KEY This is a unique identifier within the S5 database.

EVIDENCE This is an evidence code showing how the information in the term was curated, i.e. the level of evidence supporting the phenotype annotation. The codes are the same as those used in the Human Phenotype Ontology database for compatibility (see http://www.human-phenotype-ontology.org/contao/index.php/annotation-guide.html). At the moment this only takes one value, 'ITM' stands for 'Inferred by Text Mining'. Other codes will include 'IEA' for 'Inferred from Electronic Annotation', 'PCS' for 'Published Clinical Study', 'TAS' for 'Traceable Author Statement'.

DATE The date on which the term annotation was created. The format is YYY.MM.DD.

Element: qualifierList

This data element is optional and will in the future encode all possible seen qualifiers that are encoded within the PATO 'qualitative:intensity:intensity' subtree, e.g. 'mild','moderate','remittent','severe'. PATO stands for Phenotypic Attribute and Trait Ontology.

Element: Link

The data in this field represents a link to an external annotation about the term or part of the term. This is important for grounding the semantics of the term in widely used external vocabularies, to allow interoperability and reasoning.

Attributes for Term include:

text This is the part of the term about which the annotation refers to

ID This is the URL (Universal Resource Indicator) for the external vocabulary entry

evidence This is the name of the agent who provided the link, e.g. 'NCBO Annotator' or 'Bio-LarK'

Element: Tree

The data in the Tree element has been provided by parsing the term in its original context using the MCCJ parser (McClosky Charniak Johnson parser). The tree element is a grammatical phrase structure tree with lexical and syntactic nodes (e.g. JJ stands for Adjective and CC stands for Conjunction).

Element: associatedDisorder

After discovering phenotype candidates we applied a filtering step to verify them through association with human disorders gathered from the Online Mendelian Inheritance of Man database. We applied the R package's Apriori algorithm for identifying disorder-phenotype rules. Association rule (AR) mining attempts to discover rules between frequently co-occurring items in a transaction data set. The set of OMIM disorders and their synonyms was obtained from MEDIC. PMIDs are used to label the transaction items and are found for each phenotypes and disorder by querying the PMC E-utils RESTful Web Service. We applied Apriori using a set of parameters (support, confidence, minimum length, target) so that we retained only those association rules with carinality of 2, i.e. phenotype → disorder. The results for each phenotype are recorded in the associatedDisorder element.

Each associatedDisorder element consists of zero or more disorder elements describing the discovered OMIM association.

Attributes for associatedDisorder include:

source This is the source of evidence about the association. At the moment this takes only the value 'apriori'.

min_supp This is the value of minimum support used in the Apriori algorithm

min_conf This is the value of minimum confidence used in the Apriori algorithm

df This is the number of citations where the association between the phenotype and disorder could be found, i.e. the number of disorder elements contained in the associatedDisorder element.

Note that minlen and maxlen attributes were both set to 2 within Apriori but are not recorded in the XML data.

Element: disorder

Each disorder element consists of the name of the disorder and its OMIM identifier.

Attributes for disorder include:

supp The level of support Apriori found for the phenotype-disorder association

conf The level of confidence Apriori found for the phenotype-disorder association

lift The level of lift Apriori found for the phenotype-disorder association

pval The p-value Apriori found for the phenotype-disorder association using a Fisher's exact test.

Element: name

The name element corresponds to an entry in the DiseaseName element in the Comparative Toxicogenomics (CTD) database at http://ctdbase.org (Mount Desert Island Biological Laboratory).

Element: omim_id

The omim_id entry corresponds to the OMIM unique identifier for the disorder concept.

Element: fullTextList

This element contains zero or more links to literature citations where the phenotype term has been found through a fielded search of full text articles in the PubMed Central database. The maximum number of returned citations was bounded at 10,000. In practice the number of phenotype terms which reach this limit is quite small (<5%).

Attributes for fullTextList include:

source The source of evidence for the full text citation – this only takes one value at the moment which is 'eutils', i.e. the PubMed Central E-utilities Web interface (see http://www.ncbi.nlm.nih.gov/books/NBK25499/).

df The number of documents returned by the source about the phenotype annotation

retmax The maximum number of documents to be returned by the source

Element: ID

The ID contains the PubMed Identifier (http://www.nlm.nih.gov/bsd/disted/pubmedtutorial/020_830.html) of the literature citation where the phenotype term was found.

Element: abstractList

This element contains zero or more links to literature citations where the phenotype term has been found through a fielded search of abstracts in the PubMed Central database. The maximum number of returned citations was bounded at 10,000. In practice the number of phenotype terms which reach this limit is quite small (<5%).

Attributes for abstractList include:

source The source of evidence for the full text citation – this only takes one value at the moment which is 'eutils', i.e. the PubMed Central E-utilities Web interface (see http://www.ncbi.nlm.nih.gov/books/NBK25499/).

df The number of documents returned by the source about the phenotype annotation

retmax The maximum number of documents to be returned by the source

Element: ID

The ID contains the PubMed Identifier (http://www.nlm.nih.gov/bsd/disted/pubmedtutorial/020_830.html) of the literature citation where the phenotype term was found.

REST search

A search request can be executed purely using a URI by providing request parameters to the PhenoMiner server.

e.g. http://phenominer.mml.cam.ac.uk/search.html?q=*:*

The parameters allowed in the URI are:

ID	Name	Desciption
1	**q**	The query string (See more section 3 Query syntax)
	**Example: http://phenominer.mml.cam.ac.uk:8080/phenominer/phenotype/_search?q="abnormal kidney"**	
2	**fields**	The selective stored fields of the document to return for each hit, comma delimited.
	**Example: http://phenominer.mml.cam.ac.uk:8080/phenominer/phenotype/_search?q="abnormal kidney"&fields=id,key**	
3	**size**	The number of hits to return. Defaults to 10.
	**Example: http://phenominer.mml.cam.ac.uk:8080/phenominer/phenotype/_search?q="abnormal kidney"&size=4**	
4	**pretty**	Tells system to return pretty-printed JSON/XML results
	**Example: http://phenominer.mml.cam.ac.uk:8080/phenominer/phenotype/_search?q="abnormal kidney"&pretty**	

Query syntax

Query	Decription	Example
**Normal**	Normal query and phrase/exact query	q="abnormal heart"
**Match all**	Match all data in database	q=*:*<br>q=*
**Field**	Search on a field	q=id:"abnormal heart"
**Multi fields**	Search on multi fields	q=id:* AND link.text:sudden

Web-based GUI

You can type your phenotype terms query to input box. The query syntax is similar to the syntax shown above.

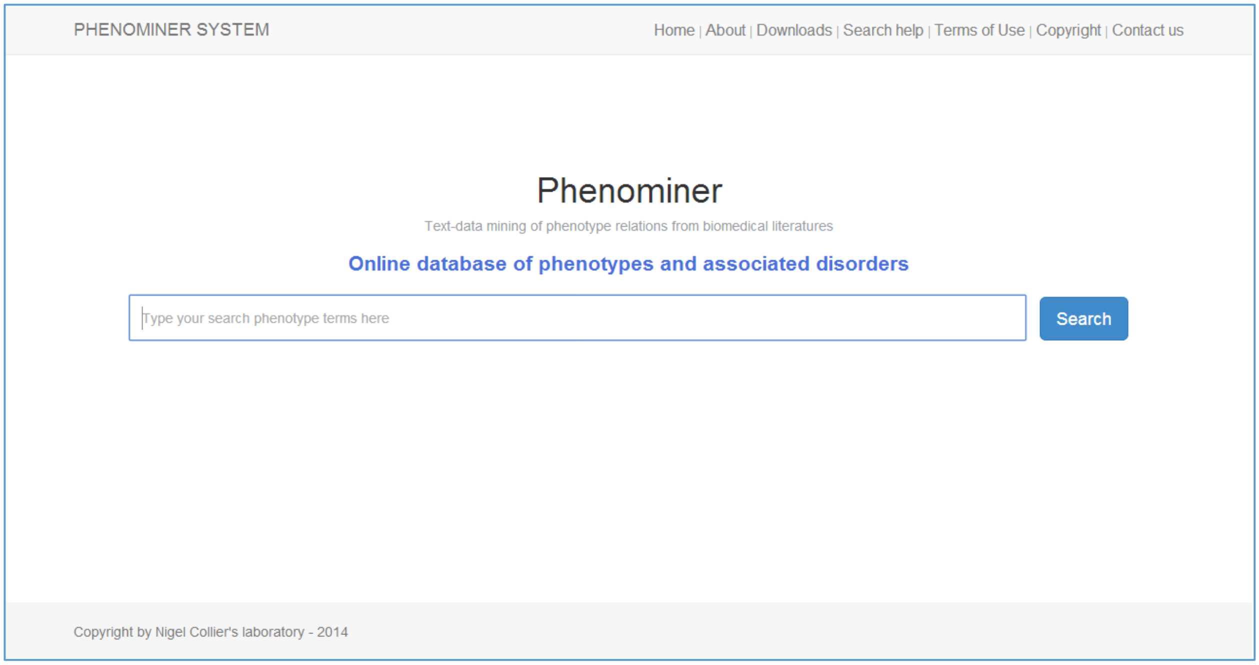

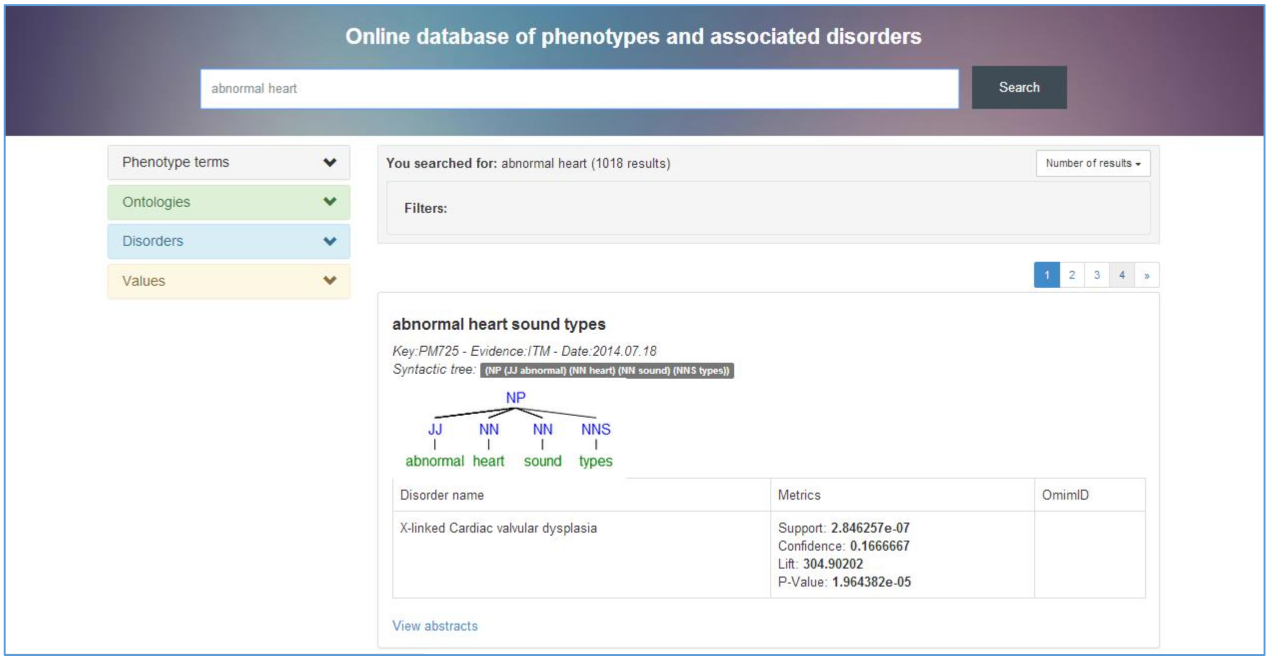

The PhenoMiner system shows some fields related to each phenotype entity:

- ID
- Key
- Evidence
- Date
- Syntactic phrase structure tree
- List of terms (related to biomedical ontologies)
- List of disorders (related to phenotype entity)
- List of Pubmed Central abstracts

abnormal kidney function

Key:PM810 - Evidence:ITM - Date:2014.07.18
Syntactic tree: (NP (JJ abnormal) (NN kidney) (NN function))

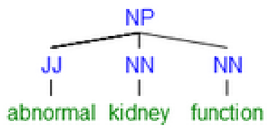

Term	Ontology	Evidence
abnormal	Systematized Nomenclature of Medicine - Clinical Terms	NCBO Annotator
abnormal	Phenotypic Quality Ontology	NCBO Annotator
abnormal	Mammalian Phenotype Ontology	NCBO Annotator
kidney	Systematized Nomenclature of Medicine - Clinical Terms	NCBO Annotator
kidney	Foundation Model of Anatomy	NCBO Annotator
kidney	Mammalian Phenotype Ontology	NCBO Annotator
function	Systematized Nomenclature of Medicine - Clinical Terms	NCBO Annotator
function	Mammalian Phenotype Ontology	NCBO Annotator
abnormal kidney	Human Phenotype Ontology	Bio-LarK

Disorder name	Metrics	OmimID
Essential Hypertension	Support: **8.538771e-07**<br>Confidence: **0.1111111**<br>Lift: **39.53778**<br>P-Value: **1.132452e-08**	
Polycystic Kidney Diseases	Support: **1.423129e-06**<br>Confidence: **0.1851852**<br>Lift: **31.75107**<br>P-Value: **8.619740e-13**	OMIM:173900

View abstracts

You can select the number of results using the drop down list:

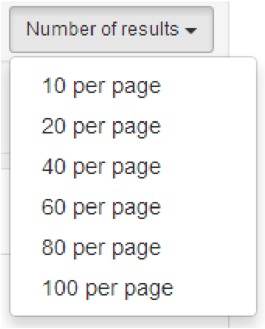

Then use paging to view the results:

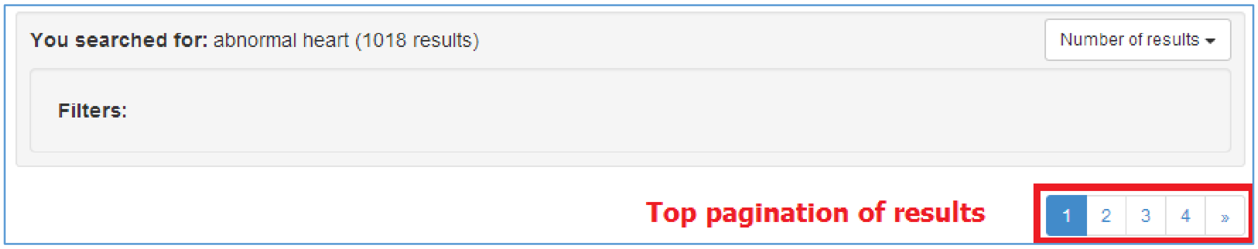

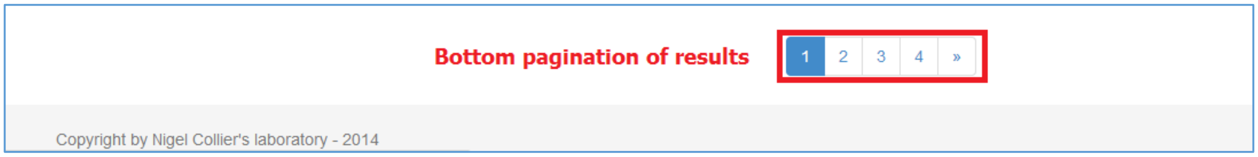

The PhenoMiner system will show list of terms (related to query) from Phenotype field, Ontology field, Disorder field and P-Value field. You can select terms in each fields to refine results.

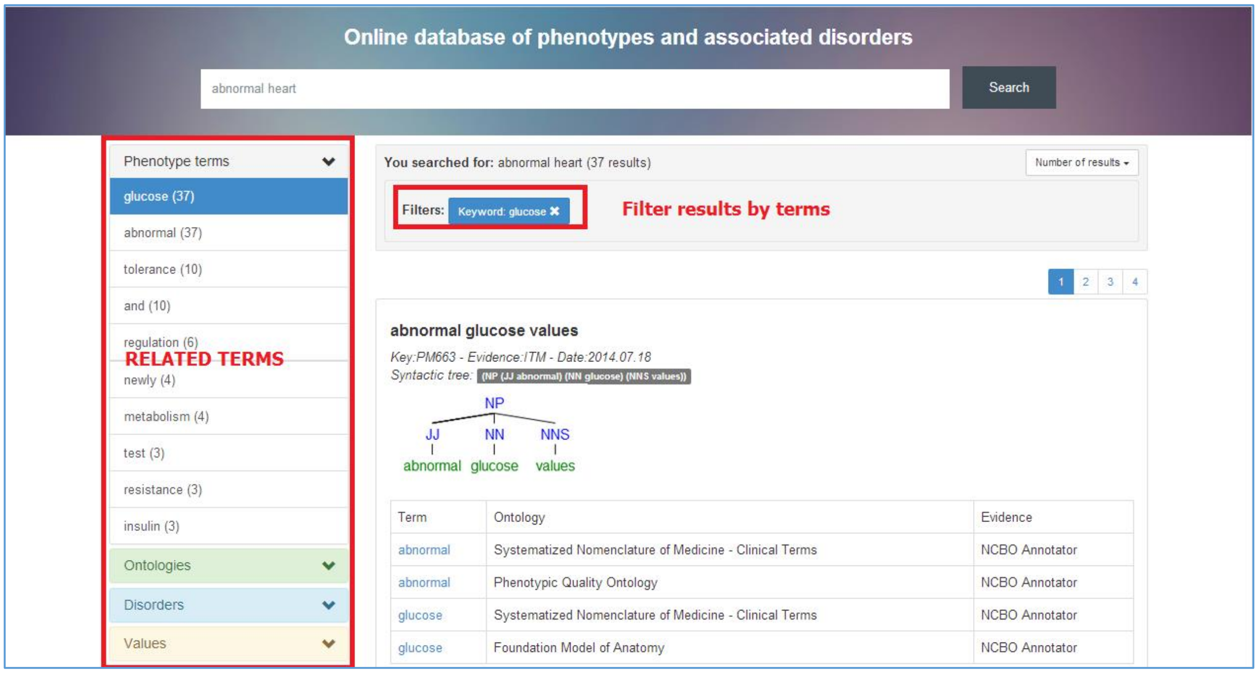

To know more about the PhenoMiner database, you can select from the links at the top of the page:

Home | About | Downloads | Search help | Terms of Use | Copyright | Contact us

- About page: Basic introduction to the PhenoMiner project and database.
- Downloads page: You can find various resources related to the PhenoMiner project such as an annotated corpus, published papers and links to GitHub, Zenodo and Twitter.
- Search help page: A link to this file!
- Terms of Use page: conditions for accessing our system
- Copyright page: Information about copyright
- Contact us page: If you have any questions, please contact with us through information in this page.

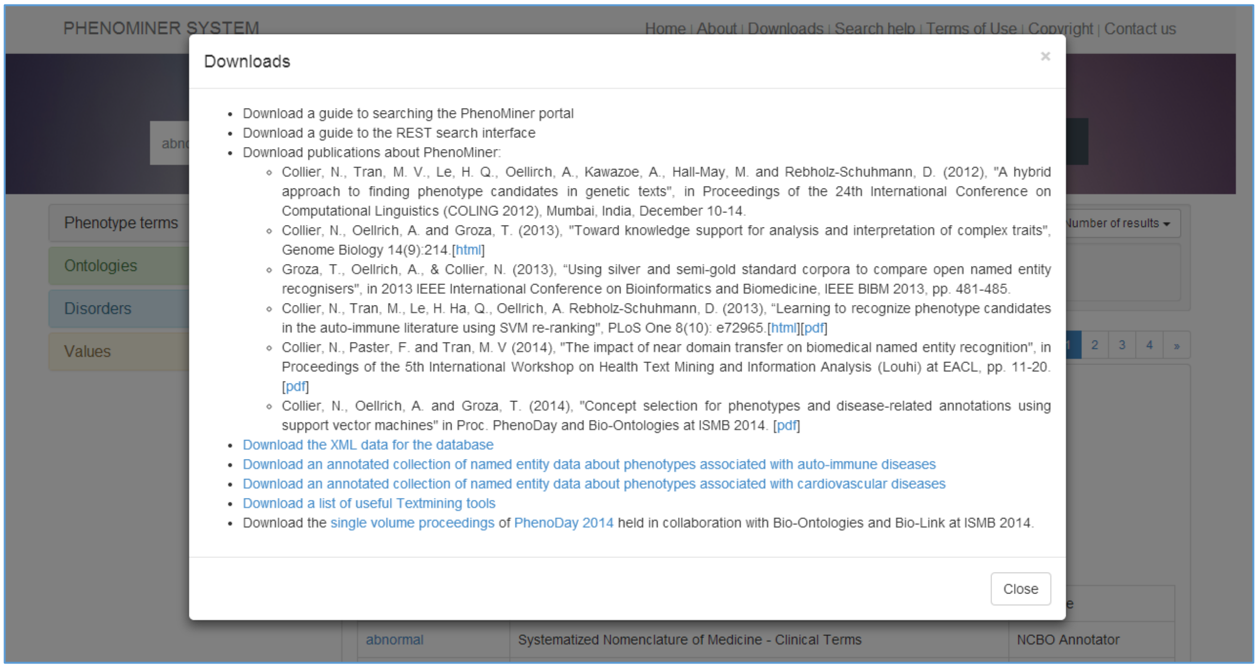

PHENOMINER SYSTEM
Home | About | Downloads | Search help | Terms of Use | Copyright | Contact us
Downloads
Download a guide to searching the PhenoMiner portal
Download a guide to the REST search interface
Download publications about PhenoMiner:
Collier, N., Tran, M. V., Le, H. Q., Oellircn, A., Kawazoe, A., Hall-May, M. and Rebholz-Schuhmann, D. (2012), "A hybrid approach to finding phenotype candidates in genetic texts", in Proceedings of the 24th International Conference on Computational Linguistics (COLING 2012), Mumbai, India, December 10-14.
Collier, N., Oellrich, A. and Groza, T. (2013), "Toward knowledge support for analysis and interpretation of complex traits", Genome Biology 14(9):214.[html]
Groza, T., Oellrich, A., & Collier, N. (2013), "Using silver and semi-gold standard corpora to compare open named entity recognisers", in 2013 IEEE International Conference on Bioinformatics and Biomedicine, IEEE BIBM 2013, pp. 481-485.
Collier, N., Tran, M., Le, H. Ha, Q., Oellrich, A. Rebholz-Schuhmann, D. (2013), "Learning to recognize phenotype candidates in the auto-immune literature using SVM re-ranking", PLoS One 8(10): e72965.[html][pdf]
Collier, N., Paster, F. and Tran, M. V (2014), "The impact of near domain transfer on biomedical named entity recognition", in Proceedings of the 5th International Workshop on Health Text Mining and Information Analysis (Louhi) at EACL, pp. 11-20. [pdf]
Collier, N., Oellrich, A. and Groza, T. (2014), "Concept selection for phenotypes and disease-related annotations using support vector machines" in Proc. PhenoDay and Bio-Ontologies at ISMB 2014. [pdf]
Download the XML data for the database
Download an annotated collection of named entity data about phenotypes associated with auto-immune diseases
Download an annotated collection of named entity data about phenotypes associated with cardiovascular diseases
Download a list of useful Textmining tools
Download the single volume proceedings of PhenoDay 2014 held in collaboration with Bio-Ontologies and Bio-Link at ISMB 2014.
Close
Phenotype terms
Ontologies
Disorders
Values
Number of results
2
3
4
»
abnormal
Systematized Nomenclature of Medicine - Clinical Terms
NCBO Annotator
