# Supplementary material for: PhenoMiner: from text to a database of phenotypes associated with OMIM diseases
Source: Database (Oxford). 2015 Oct 27;2015:bav104. doi: 10.1093/database/bav104 (PMC4622021; doi:10.1093/database/bav104)
Supplement: Supplementary Data [file supp_bav104_S4.pdf]

Supplementary Material S4:

List of Abbreviations

DOID	Human Disease Ontology
EPR	Electronic Patient Record
EQ	Entity Quality representation
FMA	Foundation Model of Anatomy
GO	Gene Ontology
HP	Human Phenotype Ontology
MA	Mouse Adult Gross Anatomy Ontology
MEDIC	Merged Disease Vocabulary
MGD	Mouse Genome Database
MP	Mammalian Phenotype Ontology
NCBO	National Center for Biomedical Ontology
NER	Named Entity Recognition
OMIM	Online Mendelian Inheritance of Man database
ORDO	Orphanet Rare Disease Ontology
PATO	Phenotypic Attribute and Trait Ontology
PMID	PubMed Identifier
PM	PhenoMiner
UMLS	Unified Medical Language System
XML	Extensible Markup Language
